# MicroRNA-30b controls endothelial cell capillary morphogenesis through regulation of transforming growth factor beta 2

**DOI:** 10.1371/journal.pone.0185619

**Published:** 2017-10-04

**Authors:** Grant A. Howe, Kayla Kazda, Christina L. Addison

**Affiliations:** 1 Cancer Therapeutics Program, Ottawa Hospital Research Institute, Ottawa, ON, Canada; 2 Department of Biochemistry, Microbiology and Immunology, University of Ottawa, Ottawa, ON, Canada; 3 Department of Medicine, University of Ottawa, Ottawa, ON, Canada; Universitat des Saarlandes, GERMANY

## Abstract

The importance of microRNA (miRNA) to vascular biology is becoming increasingly evident; however, the function of a significant number of miRNA remains to be determined. In particular, the effect of growth factor regulation of miRNAs on endothelial cell morphogenesis is incomplete. Thus, we aimed to identify miRNAs regulated by pro-angiogenic vascular endothelial growth factor (VEGF) and determine the effects of VEGF-regulated miRNAs and their targets on processes important for angiogenesis. Human umbilical vein endothelial cells (HUVECs) were thus stimulated with VEGF and miRNA levels assessed using microarrays. We found that VEGF altered expression of many miRNA, and for this study focused on one of the most significantly down-regulated miRNA in HUVECs following VEGF treatment, miR-30b. Using specific miRNA mimics, we found that overexpression of miR-30b inhibited capillary morphogenesis *in vitro*, while depletion of endogenous miR-30b resulted in increased capillary morphogenesis indicating the potential significance of down-regulation of miR-30b as a pro-angiogenic response to VEGF stimulation. MiR-30b overexpression in HUVEC regulated transforming growth factor beta 2 (TGFβ2) production, which led to increased phosphorylation of Smad2, indicating activation of an autocrine TGFβ signaling pathway. Up-regulation of TGFβ2 by miR-30b overexpression was found to be dependent on ATF2 activation, a transcription factor known to regulate TGFβ2 expression, as miR-30b overexpressing cells exhibited increased levels of phosphorylated ATF2 and depletion of ATF2 inhibited miR-30b-induced TGFβ2 expression. However, miR-30b effects on ATF2 were indirect and found to be via targeting of the known ATF2 repressor protein JDP2 whose mRNA levels were indirectly correlated with miR-30b levels. Increased secretion of TGFβ2 from HUVEC was shown to mediate the inhibitory effects of miR-30b on capillary morphogenesis as treatment with a neutralizing antibody to TGFβ2 restored capillary morphogenesis to normal levels in miR-30b overexpressing cells. These results support that the regulation of miR-30b by VEGF in HUVEC is important for capillary morphogenesis, as increased miR-30b expression inhibits capillary morphogenesis through enhanced expression of TGFβ2.

## Introduction

Angiogenesis is the process of new blood vessel growth from pre-existing vessels. It is a complex tightly regulated process that involves a number of coordinated steps for vessel formation to occur. A number of factors that promote angiogenesis have been identified, with vascular endothelial growth factor being the best characterized. In addition to pro-angiogenic factors, a number of molecules have been shown to inhibit the angiogenic process, and it is the balance between these anti-angiogenic factors and pro-angiogenic factors that dictate whether new vessel formation will occur.

In addition to the well-studied proteinacious pro- and anti-angiogenic factors, the importance of microRNAs (miRNA) to angiogenesis has been more recently suggested from earlier studies demonstrating the importance of the miRNA processing enzyme Dicer to embryonic vascular development [1], *in vitro* angiogenic processes such as capillary morphogenesis and sprouting [2], and *in vivo* vessel formation in response to angiogenic stimuli [3]. MiRNAs are small RNA molecules of ~22 nucleotides in size. They are found in almost every living system, from viruses to plants to animals, and are known to regulate message RNA (mRNA) levels via their ability to bind to target mRNA and either sequester it from being translated into protein or cause it to be degraded [4,5]. Expression profiling of human umbilical vein endothelial cells (HUVEC) [6] and subsequently other endothelial cell types [7] has provided insight into the importance of individual miRNA expression patterns to endothelial cell biology. Since those initial studies, roles for individual miRNAs in angiogenic processes are increasingly being identified with both pro- [8–13] and anti-angiogenic [14–17] effects being observed. However, many of these identified miRNAs have yet to be fully described in terms of the mechanism by which they regulate angiogenesis and many more remain as yet unstudied. As miRNAs contribute to a number of disease states in which angiogenesis also plays a significant role, including cancer [18], cardiovascular disease [19], liver disease [20] and rheumatoid arthritis [21], new studies are attempting to assess the feasibility of manipulating miRNA expression to combat such diseases [22,23]. Thus, a better understanding of the roles of individual specific miRNAs is vitally important for determining the feasibility of manipulating such miRNAs for therapeutic purposes to combat pathological angiogenesis.

It is well known that angiogenesis is controlled by a balance of factors that promote angiogenesis and those that inhibit the process. VEGF is one of the most potent pro-angiogenic factors identified to date. A number of studies have recently shown that VEGF production can be regulated by numerous miRNA [24–29], again highlighting the importance of miRNA to the angiogenic process. However, there is a lack of information regarding whether or not VEGF itself is capable of regulating the transcriptional production of miRNA which in turn play a role in angiogenesis. As such, we were interested to determine whether VEGF stimulation of endothelial cells resulted in altered miRNA expression and whether these altered miRNA contributed to vessel formation. Following VEGF stimulation, endothelial expression of miRNA was assessed using Affymetrix miRNA expression arrays. We identified a number of VEGF-regulated miRNA and focused our further study of the role of one of the most highly downregulated miRNA, namely miR-30b.

MiR-30b is a member of the five-member miR-30 family of miRNAs which are encoded over 6 genes and expressed from 4 distinct transcripts [30]. The miR-30 family of miRNA are highly conserved across species and share the same seed sequence. MiR-30b has not been well studied to date, but has been shown to play a role in myogenesis [31] and osteoblastogenesis [32,33]. However, overexpression of miR-30 family members in zebrafish models suggest they promote angiogenesis [34,35], which would not be in line with our findings that it is suppressed by the potent pro-angiogenic factor VEGF. As such we wished to further confirm our initial findings of VEGF regulation of miR-30b, and determine the outcome of modulation of miR-30b expression in human endothelial cells on capillary morphogenesis. We found that miR-30b overexpression in HUVEC is associated with impaired capillary morphogenesis in part through autocrine regulation of TGFβ2 expression. We further found this is due in part to the ability of miR-30b to down-regulate expression of Jun dimerization protein 2 (JDP2), a repressor of the activating transcription factor 2 (ATF2) protein which is known to promote transcription of TGFβ2 [36]. This study further implicates TGFβ2 as a negative regulator of angiogenic processes and provides important insight regarding endothelial cell response to pro-angiogenic VEGF stimulation via miRNA regulation of transcription factor activity.

## Materials and methods

### Antibodies and growth factors

Primary antibodies used were: TGFβ2 (V, SC-90), ATF-2 (C-19, SC-187), and phospho-ATF-2 (F-1, SC-8398) from Santa Cruz Biotechnology (Santa Cruz, CA), phospho-Smad2 (S465/467) from Cell Signaling Technology (3101; Danvers, MA), Smad2 from Invitrogen (511300; Carlsbad, CA), β-Actin (clone AC-74) from Sigma-Aldrich (A5316; St. Louis, MO), anti-TGFβ2 neutralizing antibody (AB-12-NA) and Normal Rabbit IgG (AB-105-C) from R&D Systems (Minneapolis, MN). Secondary antibodies used were: goat anti-mouse IgG horse radish peroxidase (HRP) conjugate and goat anti-rabbit IgG HRP conjugate, both from Calbiochem (EMD Biosciences, La Jolla, CA). Recombinant human VEGF_165_ was purchased from R&D Systems (Minneapolis, MN). Recombinant Human TGFβ2 (100-35B) was from Peprotech (Quebec, QC). Avastin® (DIN 02270994) was from Roche (Mississauga, ON) and was used at a concentration of 1 μg/ml.

### Cell culture

Human umbilical vein endothelial cells (HUVECs) were purchased from Lonza (C2517A; Walkersville, MD) and grown in EGM-2 media [EBM-2 basal medium (CC-3156) supplemented with EGM-2 SingleQuot kit supplement and growth factors (CC-4176)] also from Lonza. Cells were routinely passaged at 80–90% confluence and used for experiments at passage 6 through 10. Cells were maintained at 37°C in 5% CO_2_. All experiments were performed in EGM-2 unless otherwise noted. For serum starvation, HUVECs were incubated in MCDB 131 Medium (Gibco by Life Technologies; Carlsbad, CA) supplemented with L-Glutamine (GlutaMAX-I; Gibco by Life Technologies, Carlsbad, CA) and 0.5% fetal bovine serum (FBS: Medicorp, Montreal, QC) for 16–20 hours, with additional time under starvation as required under specific experimental conditions.

### SiRNA transfection

ATF2 ON-TARGET siRNA (ATF2 siRNA 1; MQ-009871-00) and control non-targeting siRNA (siControl Non-Targeting siRNA #1; D-001210-01) were purchased from Dharmacon (Lafayette, CO). ATF2 Silencer siRNA (ATF2 siRNA 2; AM16708A) was purchased from Ambion (Ambion, ThermoFisher Scientific, Burlington ON). For silencing of ATF2, both ATF2 siRNAs and control siRNA were used at concentrations of 5 or 50 nM. HUVECs were transfected at 80% confluence in Opti-MEM® I reduced serum medium using Oligofectamine Transfection Reagent (Invitrogen, Carlsbad, CA), according to the manufacturer’s protocol.

### Transfection of microRNA mimics and hairpin inhibitors

HUVECs were seeded onto either 6 cm or 10 cm tissue culture plates at 4 x 10^5^ cells or 1 x 10^6^ cells, respectively, and allowed to adhere overnight. Transfection of cells with miRIDIAN microRNA Mimics or miRIDIAN microRNA Hairpin Inhibitors (all from Thermo Fisher Scientific Inc., Waltham, MA) was achieved with Oligofectamine Transfection Reagent (Invitrogen, Carlsbad, CA), according to the manufacturer’s instructions. Mimics used were: miRIDIAN microRNA Mimic Negative Control #1 and miRIDIAN microRNA hsa-miR-30b-5p mimic. Hairpin inhibitors used were: miRIDIAN microRNA Hairpin Inhibitor Negative Control #1 and miRIDIAN microRNA hsa-miR-30b-5p hairpin inhibitor.

### Western blotting

Equal amounts of total protein per sample were diluted with NuPAGE® LDS Sample Buffer (Novex, Life Technologies, Carlsbad, CA) and reduced with dithiothreitol (DTT). Electrophoresis was performed with NuPAGE® Novex® 4–12% Bis-Tris Gels (Novex, Life Technologies, Carlsbad, CA) and proteins were transferred to Hybond-C Extra nitrocellulose membrane (Amersham Biosciences, GE Healthcare, Piscataway, NJ). The following concentrations of primary antibody were used: TGFβ2 (1:200), phospho-ATF-2 (1:200), ATF-2 (1:200), phospho-Smad2 (1:1000), Smad2 (1:1000), β-actin (1:7000). Following primary antibody incubation, membranes were incubated in appropriate secondary antibodies and images were developed in Immobilon Western Chemiluminescent HRP Substrate (EMD Millipore, Billerica, MA) prior to visualization using the GeneGnome detection system (Syngene, Frederick, MD).

### RNA isolation

HUVECs were washed once with PBS, followed by the addition of 700 μl QIAzol lysis reagent (Qiagen, Germantown, MD). Cell lysate was frozen at -80°C until processing for total RNA including miRNA with the miRNeasy Mini Kit (Qiagen, Germantown, MD) according to the manufacturer’s protocol. RNA was dissolved in sterile nuclease free water and stored at -80°C.

### Quantitative RT-PCR

Reverse transcription was performed with Moloney murine leukemia virus (M-MLV) reverse transcriptase (RT) (Invitrogen by Life Technologies, Carlsbad, CA) according to the manufacturer’s instructions. PCR was performed as individual reactions with gene specific primers and RT^2^ SYBR Green ROX™ qPCR Mastermix (Qiagen, Germantown, MD). PCR was performed with a 7500 Fast Real-Time PCR System (Applied Biosystems by Life Technologies, Carlsbad, CA). The amount of RNA in each sample was normalized to β-actin levels within that sample. Relative expression was determined via delta-delta-Ct method with values displayed as 2^-ΔΔCt^. The primer sets used were: β-actin (forward: CCAACCGCGAGAAGATGA; reverse: CCAGAGGCGTACAGGGATAG), TGFβ1 (forward: CACGTGGAGCTGTACCAGAA; reverse: CAGCCGGTTGCTGAGGTA), TGFβ2 (forward: CCAAAGGGTACAATCCAC; reverse: CAGATTCTGGATTTATGGTATT), ATF2 (forward: TTTGGTCCAGCACGTAATGA; reverse: CAAACCCACTTCTTCACAGTTTT), JDP2 (forward: TTTGCAGGGAGGTGCTCT; reverse: GATCTGCCCAGGCATCATA).

### MicroRNA expression analysis

For microarray analysis, RNA was labeled using the Flashtag HSR biotin labeling kit and used to probe Affymetrix GeneChip Human miRNA 2.0 arrays (Affymetrix, Santa Clara CA) as per manufacturer’s instructions in the Stemcore Core Facility at Ottawa Hospital Research Institute. Analysis of gene expression following modulation of miR-30b levels was performed with an Affymetrix GeneChip Human Gene 1.0 ST array also at Stemcore Facility according to manufacturer’s instructions. For quantitative RT-PCR of miRNA, RNA samples were diluted to a concentration of 5 ng/μl in sterile nuclease free water and reverse transcription of desired mature miRNAs was performed, with up to four miRNA-specific primers in the same reaction, using TaqMan MicroRNA Assays (Applied Biosystems by Life Technologies, Carlsbad, CA) specific for individual miRNAs and the TaqMan MicroRNA Reverse Transcription Kit (Applied Biosystems by Life Technologies, Carlsbad, CA), following the manufacturer’s protocol. PCR was performed with the TaqMan MicroRNA Assay PCR primer for the desired miRNA and the TaqMan 2X Universal PCR Master Mix (Applied Biosystems by Life Technologies, Carlsbad, CA). PCR was performed with a 7500 Fast Real-Time PCR System (Applied Biosystems by Life Technologies, Carlsbad, CA). Primers used were as follows: hsa-miR-30b (assay # 000602) hsa-miR-103 (assay # 000439), RNU24 (assay # 001001) and hsa-miR-21 (assay # 000397). Gene expression data were normalized to miR-103 unless otherwise stated. Relative expression was determined via delta-delta-Ct method with values displayed as 2^-ΔΔCt^.

### Cell viability assay

HUVECs were seeded into 6-well tissue culture plates at a density of 9 x 10^4^ cells/well in triplicate, and cells were maintained in EGM-2 growth media. To assess cell viability, cells were washed once in Hank’s Balanced Salt Solution (HBSS) to remove floating debris, and then trypsinized. Viability was assessed by trypan blue exclusion using a Vi-Cell XR cell viability analyzer (Beckman Coulter, Brea, CA) at the times indicated. For transfection experiments, HUVECs were seeded for the assay at 24 hours post transfection and time points used for viable cells counts indicate 24 hour intervals post seeding.

### Migration assay

Cell migration was assessed via scratch wound assay. HUVECs were seeded into 6-well tissue culture plates at 4.5 x 10^5^ cells/well in duplicate and allowed to grow overnight to 100% confluence. A wound of approximately 1.5 mm was made creating a gap into which cells could migrate. For transfection experiments, cells were seeded at 24 hours post transfection and wounding was performed at 48 hours post transfection. Images were taken at time of wounding and 24 hours post wounding with a Nikon Eclipse TE2000-U microscope. Migration was assessed by calculating the distance migrated from 12 total measurements taken across the wound front from each of duplicate wells for each experiment.

### Capillary morphogenesis assay (Cord formation)

The organization of HUVECs into capillary-like cord structures was assessed by plating cells onto Cultrex^®^ Basement Membrane Extract (BME, Growth factor reduced, Trevigen, Gaithersburg, MD). BME was polymerized at 37°C for 30 minutes in 24-well plates followed by incubation in EGM-2 growth media for 1–2 hours. Cells were then seeded at 5 × 10^4^ cells/well in duplicate or triplicate in EGM-2 growth media. Transfected cells were seeded onto BME at 48 hours post transfection. Twenty-four hours later, images were taken with a Nikon Eclipse TE2000-U microscope. Demarcation of each well into 4 quadrants allowed for a total of 4 images per well with the total number of capillary-like cord structures and loops counted with ImageJ software (http://imagej.nih.gov/ij/), and expressed as the average number per field of view. Cords were considered to be elongated cellular extensions and loops were identified as fully enclosed areas surrounded completely by cord structures regardless of the size of the area.

### TGFβ1 and TGFβ2 ELISAs

Quantikine ELISAs from R&D Systems (Minneapolis, MN) were used for the determination of TGFβ1 (cat# SB100B) and TGFβ2 (cat# SB250) in cell culture supernates. HUVECs were transfected with 20 nM of either control miRNA mimic or miR-30b mimic and were maintained in MCDB 131 with 0.5% FBS for 24 hours prior to collection of supernates. Cell culture supernates were collected and debris was removed through centrifugation at 1,200 x g for 5 minutes followed by storage of the supernates at -80°C. ELISAs were run according to manufacturer’s instructions in duplicate and absorbance was read at 450 nm with correction at 570 nm on a Multiskan Ascent photometer (Thermo Scientific, Rockford, IL). A standard curve was created for the determination of TGFβ concentrations by interpolation. Basal levels of TGFβ in culture media alone was subtracted as background from each cell culture sample.

### Statistical analysis

Analysis of statistical significance was performed in GraphPad Prism 3 (GraphPad Software Inc.). Comparison between two groups was performed with unpaired Student’s *t*-tests. Comparisons between multiple groups were done by ANOVA with post hoc analysis. Results were considered statistically significant at *P* < 0.05.

## Results

### VEGF regulates the expression of miR-30b

**Fig 1:**
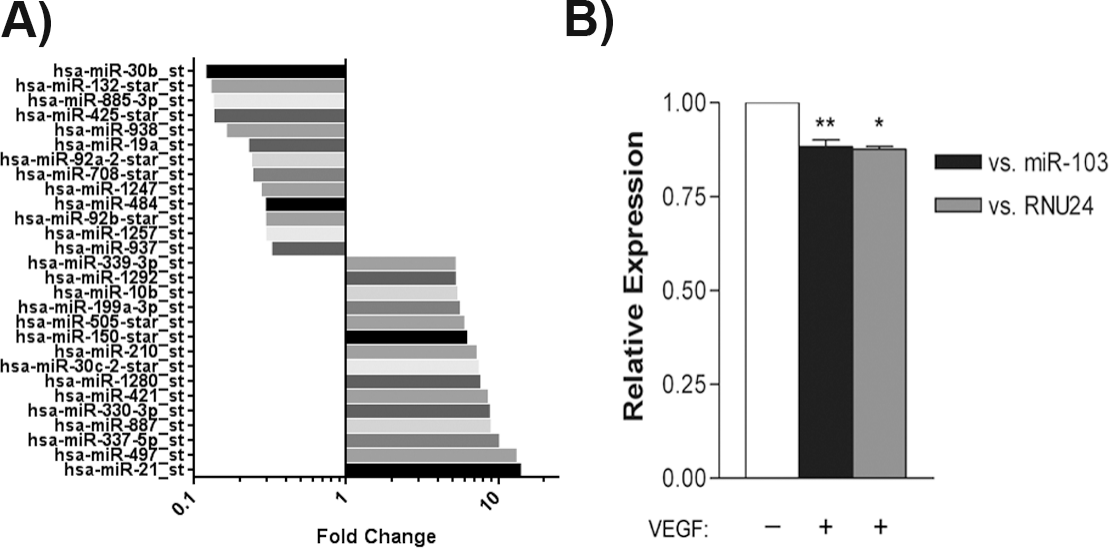
VEGF reduces miR-30b expression in HUVECs. (A) RNA was isolated from HUVEC treated with 50ng/ml of VEGF for 24h and used to profile miRNA using Affymetrix gene expression arrays. Bars represent the fold changed over unstimulated control HUVEC for some of the top targets (n = 1). (B) Cells were serum starved overnight in MCDB 131 with 0.5% FBS and stimulated with VEGF (50 ng/ml) for 24 hours. Total RNA was extracted and subjected to qRT-PCR to assess miR-30b expression. Levels of miR-30b are presented as the mean ± SEM relative to the expression of the endogenous controls miR-103 (n = 6) and RNU24 (n = 3). A statistically significant decrease in miR-30b expression is observed following VEGF stimulation. ** *P* = 0.0024 vs. miR-103; * *P* = 0.014 vs. RNU24 as determined by unpaired Student’s *t*-test.

The study of the role of individual miRNAs in angiogenesis is largely in its infancy and the regulation of miRNA expression by angiogenic growth factors is especially important to furthering our understanding of pro- and anti-angiogenic miRNAs. To this end, we examined VEGF regulation of miRNA expression in HUVECs in order to identify candidate miRNAs that may function in response to VEGF-promotion of angiogenesis. Following preliminary microarray analysis of miRNA expression in response to VEGF (S1 Table), we found a number of differentially expressed miRNA with high fold changes (Fig 1A). We identified miR-30b as a candidate miRNA for further validation. To confirm VEGF-regulation of miR-30b, HUVECs were serum starved overnight and stimulated with VEGF (50 ng/ml) for 24 hours. Expression of miR-30b in response to VEGF was compared to expression levels of both miR-103 and RNU24 as endogenous controls as our previous studies have found these are relatively invariant across different cell lines and treatments [37]. We observed that VEGF consistently and significantly downregulated miR-30b by an average of approximately 12% (Fig 1B) (*P* = 0.0024 vs. miR-103; *P* = 0.014 vs. RNU24) suggesting that miR-30b suppression may be required for VEGF-regulated angiogenic processes.

### MiR-30b regulates endothelial cell capillary morphogenesis

**Fig 2:**
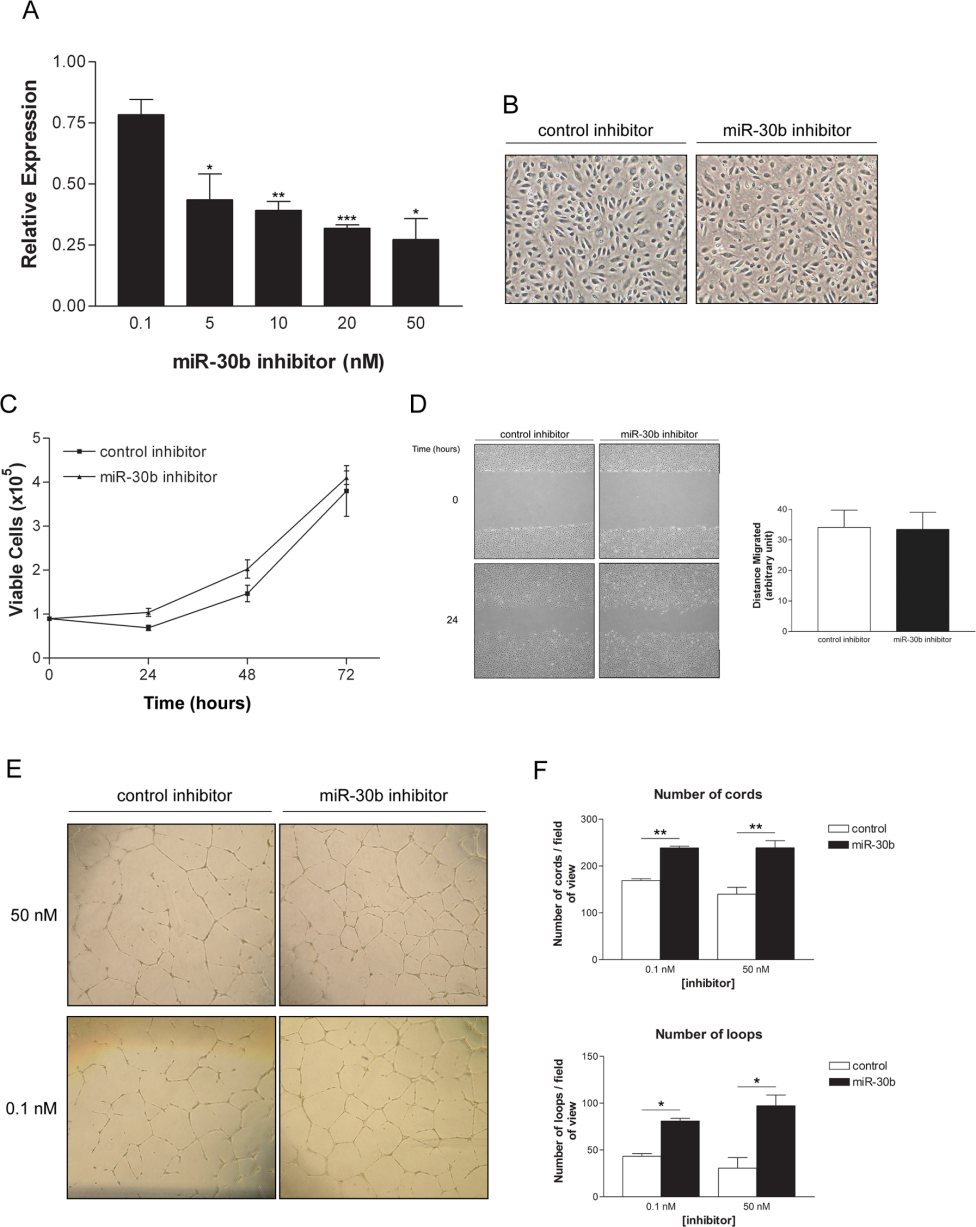
Inhibition of miR-30b enhances endothelial capillary morphogenesis. (A) HUVECs were transfected with either control miRNA inhibitor or miR-30b specific inhibitor for 48 hours. Data presented is the mean ± SEM (n = 2) normalized to the expression of miR-30b in control inhibitor transfected cells. * *P* < 0.05, ** *P* < 0.01, *** *P* < 0.001 as determined by unpaired Student’s *t*-test. (B) Morphology of HUVECs is unaffected by reduction of miR-30b using specific inhibitor (50 nM). (C) HUVECs transfected with miR-30b inhibitor (50 nM) were assessed for cell viability by trypan blue exclusion at the times indicated. Data represents the mean ± SEM (n = 2). (D) Migration of HUVECs depleted of miR-30b by specific inhibitor was assessed by scratch wound assay. Data represents the mean ± SEM (n = 2) for distance migrated after 24 hours and normalized to initial wound size. (E) Representative images of HUVEC capillary morphogenesis after 24 hours on growth factor reduced BME. Cells were transfected with control or miR-30b specific inhibitor and the total number of capillary-like cord structures and number of loops formed was assessed from duplicate wells. (F) Data represents the mean ± SEM for cells transfected with 50 nM (n = 3) or 0.1 nM (n = 2) miR-30b inhibitor. Capillary-like cord formation is significantly enhanced in cells with reduced levels of miR-30b. * *P* < 0.05, ** *P* < 0.01 as determined by unpaired Student’s *t*-test.

To study its role in angiogenesis, miR-30b expression levels were modulated through the use of miRNA inhibitors and mimics. MiR-30b levels were effectively reduced by approximately 20% at the low doses tested (0.1 nM), and up to 70% when high doses (20 nM) of specific hairpin inhibitors were tested (Fig 2A). Reduction of miR-30b levels did not alter cell morphology in monolayer (Fig 2B), nor was viability or migration affected by reduced levels of miR-30b (Fig 2C and 2D). However, depletion of miR-30b levels in untreated HUVEC was shown to cause an induction in endothelial cell capillary-like cord formation, as assessed by quantification of cord and loop number on BME (Fig 2E and 2F), suggesting miR-30b is a negative regulator of this process in endothelial cells. Importantly, we also observed a significant increase in cord formation in cells with levels of miR-30b reduction similar to those observed following VEGF stimulation (Fig 2A and 2F; 0.1 nM inhibitor concentration), indicating that relatively small decreases in the expression of miR-30b, such as those observed in response to VEGF, are biologically relevant.

**Fig 3:**
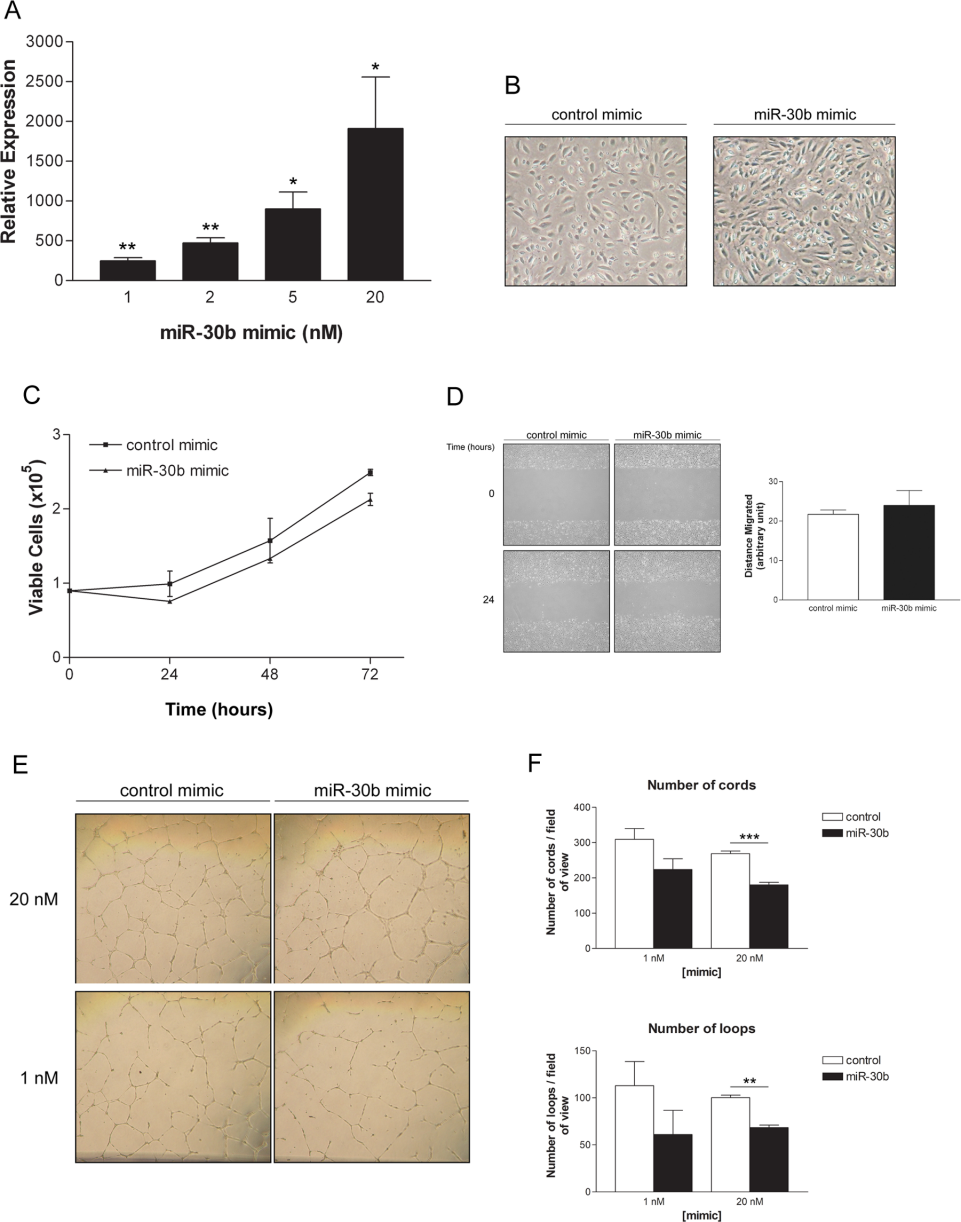
Overexpression of miR-30b reduces HUVEC capillary morphogenesis. (A) Assessment of miR-30b expression levels at 48 hours post transfection with miR-30b mimic. Data represents the mean ± SEM (n = 2) for miR-30b expression normalized to endogenous miR-30b levels in control mimic transfected cells. * *P* < 0.05, ** *P* < 0.01 as determined by unpaired Student’s *t*-test. (B) HUVEC morphology is altered following overexpression of miR-30b mimic (20 nM). (C) HUVECs overexpressing miR-30b following mimic transfection (20 nM) were assessed for cell viability by trypan blue exclusion at the indicated times. Data represents the mean ± SEM (n = 2). (D) Migration of cells transfected with miR-30b mimic (20 nM) was assessed by scratch wound assay. Data indicates the mean ± SEM (n = 2) for distance migrated after 24 hours normalized to initial wound size. (E) Capillary morphogenesis after 24 hours on growth factor reduced BME for HUVECs transfected with miR-30b mimic. (F) Data represents the mean ± SEM (n = 3) for total number of capillary-like cord structures and number of loops. Cord formation is significantly reduced in cells overexpressing miR-30b. ** *P* < 0.01, *** *P* < 0.001 as determined by unpaired Student’s *t*-test.

To confirm that miR-30b does indeed play a negative role in endothelial cell cord formation, we utilized mimics to overexpress miR-30b in HUVECs (Fig 3A). Interestingly, overexpression of miR-30b altered endothelial cell morphology, with HUVECs showing a more elongated shape (Fig 3B), reminiscent of a more mesenchymal phenotype such as that observed in the Endo-MT phenomenon [38]. However, as with depleting levels of miR-30b, overexpression of miR-30b did not affect either viability or migratory ability of HUVECs (Fig 3C and 3D). In contrast to what we observed with miR-30b reduction, overexpression of miR-30b using specific miRNA mimics reduced cord formation (Fig 3E and 3F, as assessed by both number of cords and number of loops) at varying doses. Taken together with the previous results, these findings support a negative role for miR-30b in the regulation of endothelial cell capillary morphogenesis.

### MiR-30b regulates expression of TGFβ2

**Fig 4:**
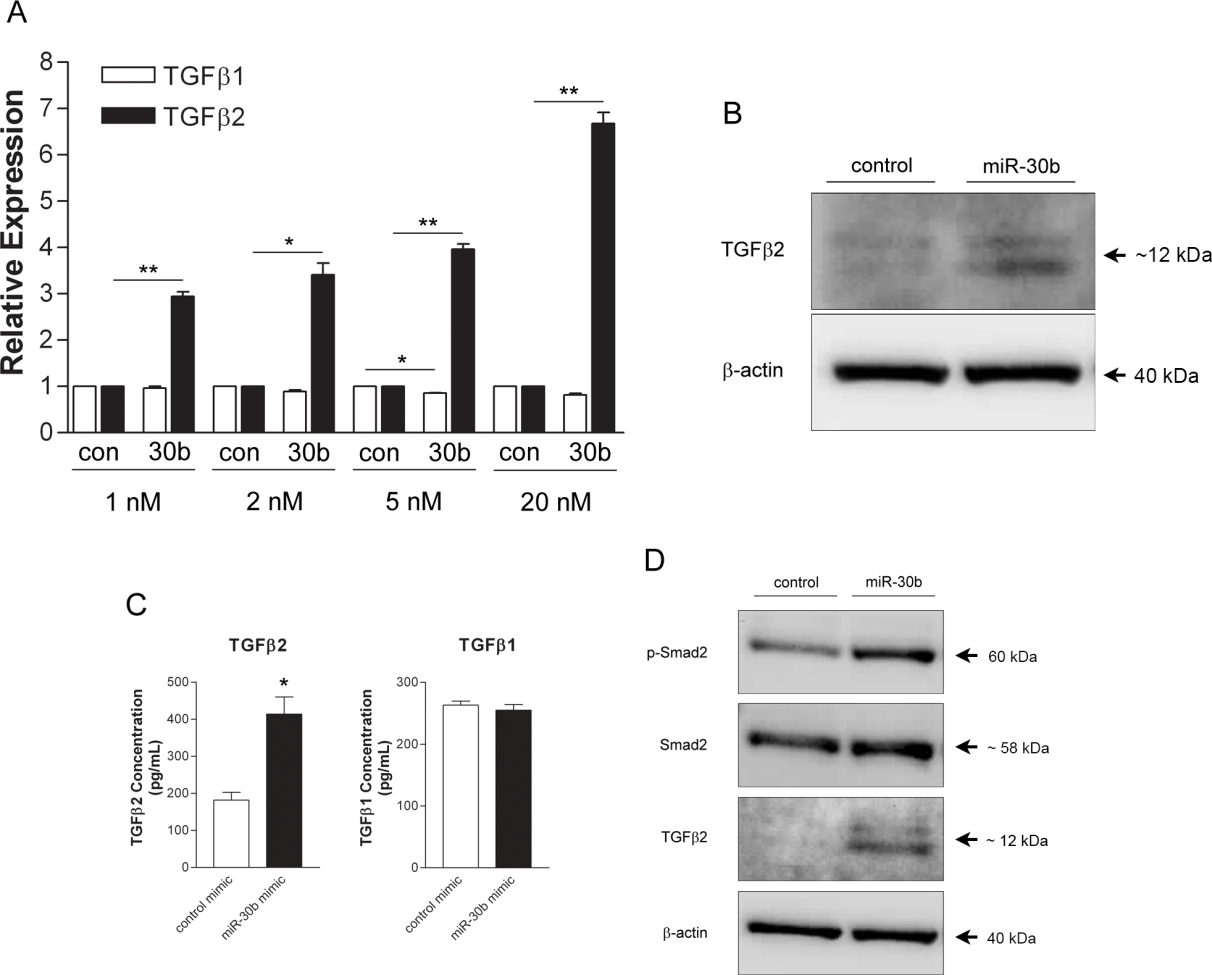
MiR-30b induces expression of TGFβ2. (A) HUVECs were transfected with either control mimic (con) or miR-30b mimic (30b) and levels of TGFβ1 and TGFβ2 mRNA were assessed by qRT-PCR. Expression levels relative to control mimic transfected cells and normalized to β-actin expression are presented as the mean ± SEM (n = 2). Overexpression of miR-30b significantly increases TGFβ2 expression. * *P* < 0.05, ** *P* < 0.01 as determined by unpaired Student’s *t*-test. (B) Cells were transfected with 20 nM of either control mimic (control) or miR-30b mimic (miR-30b) and protein lysates were collected after 48 hours for assessment of TGFβ2 protein levels by western blot. β-actin was used as endogenous control. (C) ELISAs for TGFβ1 and TGFβ2 were performed with 24 hour conditioned supernates from HUVECs transfected with 20 nM of either control or miR-30b mimic. Data represents the mean ± SEM (n = 2). Overexpression of miR-30b significantly increases TGFβ2 secretion into cell culture supernate. * *P* = 0.044 as determined by unpaired Student’s *t*-test. (D) HUVECs were transfected with 20 nM of either control mimic (control) or miR-30b mimic (miR-30b) and protein lysates were collected after 48 hours for assessment of Smad2 phosphorylation by western blot.

As our findings are in contrast to those previously observed in zebrafish that suggested miR-30b overexpression enhanced vessel formation [34], we wished to further understand the mechanism by which miR-30b could inhibit angiogenesis in human endothelial cells. In order to identify potential targets of miR-30b that could facilitate the changes in capillary-like cord formation observed, a gene expression microarray was performed on HUVECs overexpressing miR-30b using mimic constructs. Microarray analysis identified TGFβ2, among other targets, as being upregulated ~ 4-fold by miR-30b overexpression (data not shown). We chose to validate TGFβ2 as a prospective mediator of the effects of miR-30b overexpression on capillary morphogenesis as TGFβ family members have been shown to inhibit angiogenesis in some contexts [39–41], and considering the fact that it appeared, upon miR-30b upregulation, that endothelial cells gained an Endo-MT appearance which has been shown to be induced by TGFβ2 [38]. To this end, HUVECs were transfected with increasing concentrations of miR-30b mimic and expression levels of TGFβ2 were assessed by qRT-PCR. Cells overexpressing miR-30b showed a significant dose-dependent increase in levels of TGFβ2 but not the closely related family member TGFβ1 (Fig 4A), indicating the specificity of miR-30b regulation for TGFβ2. This increase in TGFβ2 mRNA also translated to increased protein expression and secretion, as TGFβ2 was elevated in both cell lysates (Fig 4B) and conditioned cell supernatants (Fig 4C) from HUVEC transfected with miR-30b mimics. TGFβ1 protein levels remained unchanged following miR-30b overexpression (Fig 4C) in line with observations of its mRNA levels. Interestingly, we also observed an increase in the phosphorylation of Smad2 (Fig 4D), a downstream signaling molecule of TGFβs, in cells overexpressing miR-30b, suggesting that increased production of TGFβ2 could be enhancing an autocrine signaling pathway in HUVECs. Taken together, these results indicate a level of regulation of TGFβ2 expression by miR-30b in HUVECs resulting in increased TGFβ2 secretion and increased signaling downstream of TGFβ receptors.

### Induction of TGFβ2 expression by miR-30b is dependent on ATF2

**Fig 5:**
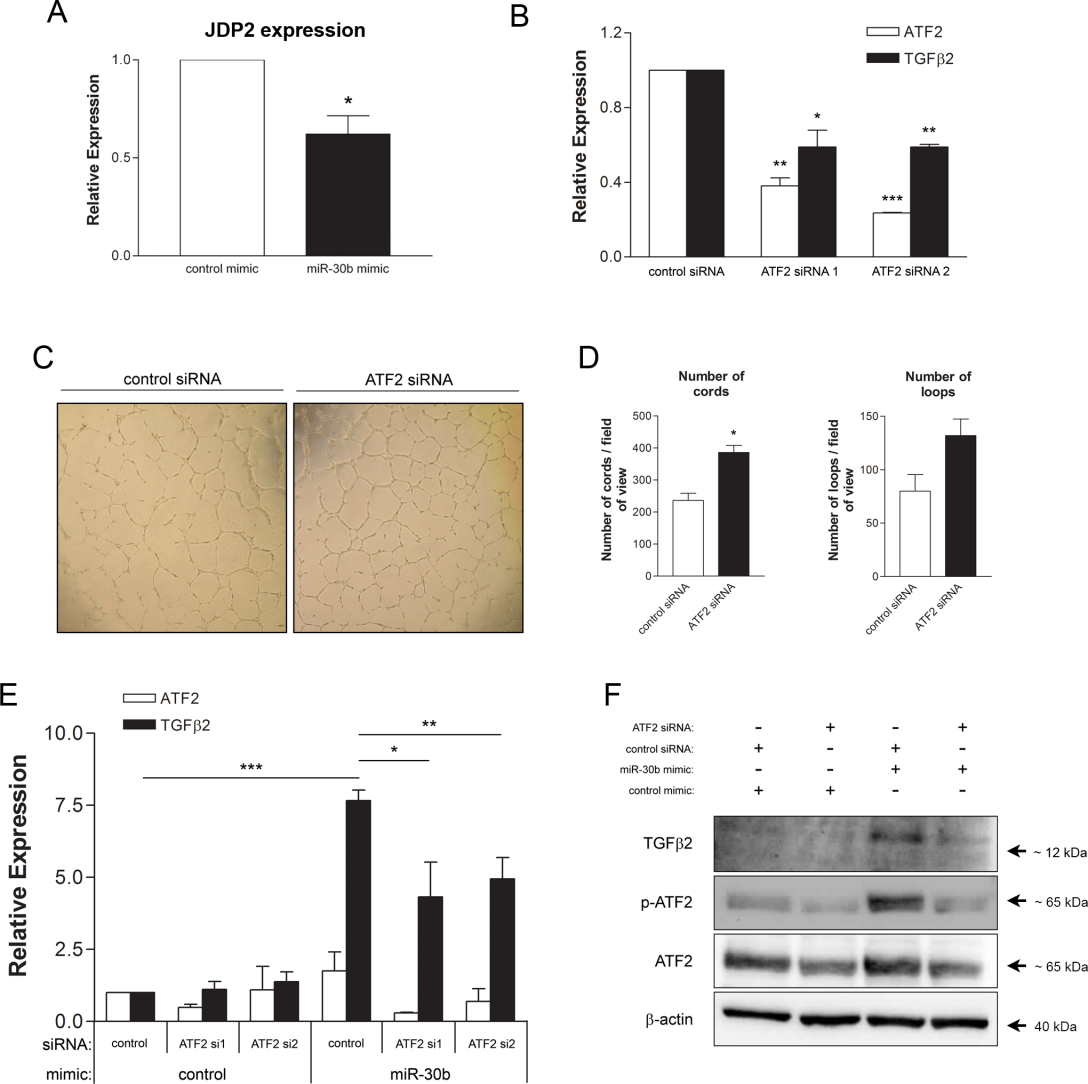
ATF2 is required for miR-30b regulation of TGFβ2 expression. (A) JDP2 mRNA expression was assessed in HUVECs transfected with miR-30b mimic (20 nM) as compared to control by qRT-PCR. Data represents the mean ± SEM (n = 3) normalized to β-actin as endogenous control. * *P* = 0.016 as determined by unpaired Student’s *t*-test. (B) HUVEC were transfected with 50 nM of either control siRNA or ATF2 siRNA 1 or 2 and RNA was isolated at 48 hours post transfection. Levels of ATF2 and TGFβ2 mRNA were assessed by qRT-PCR with β-actin as endogenous control. Data presented is mean ± SEM (n = 2). Statistically significant decreases in ATF2 and TGFβ2 expression were seen in ATF2 siRNA treated cells as compared to control siRNA treated cells. * *P* < 0.05, ** *P* < 0.01, *** *P* < 0.001 as determined by unpaired Student’s *t*-tests for each ATF2 siRNA compared to control siRNA. (C) Cells transfected with 5 nM of either control siRNA or ATF2 siRNA 1 were seeded onto growth factor reduced BME and the formation of capillary-like cord structures and number of loops was assessed after 24 hours. (D) A statistically significant increase in cord formation was observed in cells depleted of ATF2 through siRNA. Data represents the mean ± SEM (n = 2). * *P* = 0.041 as determined by unpaired Student’s *t*-test. (E) HUVECs were co-transfected with miRNA mimic (20 nM) and ATF2 siRNA 1 or 2 (50 nM) in the combinations displayed and cell lysates were collected at 48 hours post transfection and assessed for TGFβ2 mRNA expression. Data presented is mean ± SEM (n = 2). * *P* < 0.05, ** *P* < 0.01, *** *P* < 0.001 as determined by ANOVA with post hoc analysis. (F) Cells were transfected as in (E) using miRNA mimic (20 nM) and ATF2 siRNA 1 (5 nM) and serum starved overnight in MCDB 131 with 0.5% FBS prior to protein expression analysis by western blot. Data is representative of expression levels observed in two independently performed experiments.

As miR-30b positively regulates TGFβ2, hence is not directly targeting its mRNA for degradation, we hypothesized that miR-30b targets a repressor of TGFβ2 expression. Previous studies identified ATF2 as a positive transcriptional regulator of TGFβ2 expression [42], with ATF2 itself being functionally repressed by JDP2 [36], a predicted target of miR-30b (http://www.targetscan.org). Thus, we hypothesized that miR-30b could target JDP2 which would alleviate its repressive effects on ATF2, allowing for increased transcriptional activity of ATF2 thereby resulting in enhanced expression of TGFβ2. We found that overexpression of miR-30b reduced expression of JDP2 (Fig 5A) as expected based on JDP2 being a predicted target of miR-30b. SiRNA-mediated depletion of ATF2 in HUVEC, using two independent siRNA sequences, also resulted in reduced expression of TGFβ2 mRNA (Fig 5B) indicating a requirement for ATF2 in TGFβ2 gene expression. Additionally, ATF2 was shown to negatively impact endothelial capillary morphogenesis as cells depleted of ATF2 exhibited enhanced cord formation (Fig 5C and 5D), suggesting modulation of ATF2 may in fact contribute to the observed effects of miR-30b on capillary morphogenesis. To determine if ATF2 is necessary for miR-30b upregulation of TGFβ2, HUVECs were co-transfected with miR-30b mimic and ATF2 siRNA and levels of TGFβ2 were assessed. HUVECs transfected with both miR-30b mimic and ATF2 siRNA exhibited a significantly reduced level of induction of TGFβ2 mRNA (Fig 5E) and protein expression (Fig 5F) as compared to cells transfected with miR-30b mimic alone, indicating that miR-30b induction of TGFβ2 expression is dependent, at least in part, on ATF2. Interestingly, cells overexpressing miR-30b exhibited increased phosphorylated, and hence transcriptionally active ATF2 (Fig 5F), thus confirming the potential of miR-30b to regulate gene expression in HUVEC via altered transcription factor activity.

### Autocrine TGFβ2 signaling facilitates the inhibitory effects of miR-30b on capillary morphogenesis

**Fig 6:**
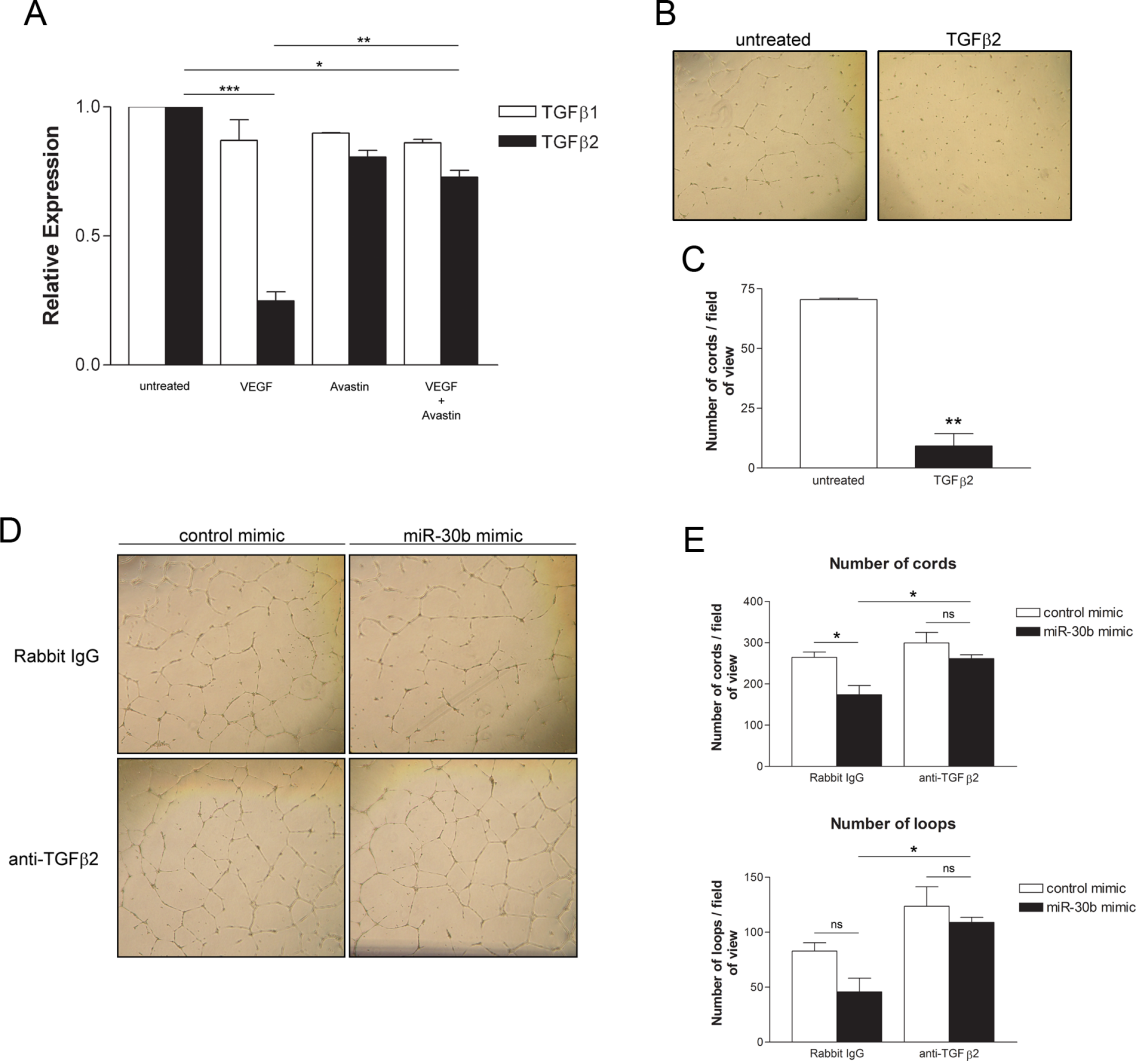
Inhibition of autocrine TGFβ2 signaling prevents reduction of capillary morphogenesis by miR-30b. (A) HUVECs were serum starved overnight in MCDB 131 with 0.5% FBS and stimulated with VEGF (50 ng/ml) in the presence or absence of Avastin (1 μg/ml) for 24 hours. Data represents the mean ± SEM (n = 2) for expression of TGFβ1 and TGFβ2 assessed by qRT-PCR relative to β-actin endogenous control. * *P* < 0.05, ** *P* < 0.01, *** *P* < 0.001 as determined by ANOVA. (B) HUVECs were treated with 5 ng/ml of TGFβ2 for 3 days prior to seeding onto growth factor reduced BME for assessment of capillary-like cord formation after 24 hours. (C) A significant decrease in cord formation is observed in the TGFβ2 treated group. Data represents the mean ± SEM (n = 2). ** *P* = 0.0072 as determined by unpaired Student’s *t*-test. (D) HUVECs transfected with 1 nM control or miR-30b mimic were treated 4 hours post transfection with 0.8 μg/ml anti-TGFβ2 neutralizing antibody or rabbit IgG. Media was refreshed after 24 hours, again with rabbit IgG or anti-TGFβ2 antibody and cells were seeded onto growth factor reduced BME 24 hours later (ie. 48 hours post transfection) in media containing rabbit IgG or anti-TGFβ2 antibody. (E) Data represents the mean ± SEM (n = 3) of the number of capillary-like cord structures or number of loops formed after 24 hours on BME. * *P* < 0.05, ns denotes not significant as determined by ANOVA with post hoc analysis.

As we have shown that miR-30b regulates the expression of TGFβ2 in endothelial cells, we wished to confirm the role of TGFβ2 in cord formation and its contribution to the negative regulatory role for miR-30b in this process. We observed that stimulation of HUVECs with VEGF decreased expression of TGFβ2 but not TGFβ1, which was prevented by concurrent treatment with the anti-VEGF monoclonal antibody Avastin (Fig 6A). Treatment of HUVECs with recombinant TGFβ2 significantly reduced cord formation by approximately 87% (Fig 6B and 6C), indicating an inhibitory role for TGFβ2 in HUVEC capillary morphogenesis in this assay system. To determine if autocrine TGFβ2 signaling induced by miR-30b contributes to the observed inhibition of capillary-like cord formation, we blocked TGFβ2 using a neutralizing antibody in miR-30b overexpressing HUVEC. In miR-30b overexpressing cells, treatment with anti-TGFβ2 antibody almost completely restored cord formation to levels seen in control miRNA transfected cells with cord and loop number significantly increased compared to miR-30b overexpressing cells treated with rabbit IgG (Fig 6D and 6E). This data suggests that negative regulation of capillary morphogenesis by miR-30b is due to enhanced TGFβ2 autocrine signaling in HUVECs, and our data support the notion that VEGF downregulates miR-30b in part to overcome the inhibitory effects of TGFβ2 production by HUVEC.

## Discussion

Growth factors regulate miRNA expression in endothelial cells [3,15,17,43–46] and in turn, miRNAs have been shown to regulate endothelial production of growth factors [17,47,48] resulting in varying levels of control over angiogenic processes. MiR-30b has been identified as a highly expressed miRNA in endothelial cells [2] with an, as yet, incompletely defined role in response to growth factor stimulation. Our results have identified miR-30b as being negatively regulated by VEGF in human umbilical vein endothelial cells. MiR-30b is located on chromosome 8q24.22 within the uncharacterized locus LOC102723694 (www.genecards.org). At present it is unclear what gene regulatory elements may be directing its expression, however a predicted promoter does appear to lie upstream of it and this promoter is predicted to have multiple binding sites for the early growth response gene 1 (Egr1) transcription factor (www.ensembl.org). Egr1 is known to be activated downstream of VEGF stimulation [49–51], and thus it is possible that it could play a role in VEGF-mediated suppression of miR-30b, however this remains to be confirmed.

The modest, although statistically significant decrease in miR-30b expression observed upon treatment of cells with VEGF was shown to be of biological significance, as when endogenous miR-30b was downregulated through the use of a hairpin inhibitor to levels similar to that observed in response to VEGF, we observed a significant increase in capillary morphogenesis. Conversely, when exogenous miR-30b was provided to endothelial cells we observed a significant decrease in capillary morphogenesis. Initially, our results seem to be in contrast to those recently reported by Bridge et al. [34], who suggested that overexpression of miR-30b resulted in increased capillary formation in vitro and in vivo. They do however show that infection of lymphatic endothelial cells with Kaposi sarcoma herpesvirus (KSHV) resulted in significant downregulation of miR-30b and miR-30c, and given that it is known that KSHV is a potent inducer of VEGF expression [52], these findings are in line with ours suggesting that VEGF stimulation downregulates miR-30b. However, they go on to show that miR-30b overexpression appears to increase capillary formation, not decrease as we have seen, and they attribute this to its ability to target DLL4. However, it remains possible that miR-30b targeting of DLL4 contributes to uncontrolled and disorganized sprouting due to loss of vessel tip cell restriction [34], which may manifest in different capillary phenotypes depending on the method of in vitro assay used, which differed in these two studies. It is also important to note that composition of endothelial media differed, and given our results suggesting that VEGF directly influences miR-30b expression, it is possible that use of alternative growth factor supplements in endothelial media could influence the observed phenotypes. It is also possible VEGF negatively regulates miR-30b in a sprouting vessel, resulting in miR-30b expression differences between tip and stalk cells as a result of increased VEGF gradients observed at the tip cell. This would theoretically result in reduced miR-30b levels in the tip cell and thus maintenance of DLL4 expression which signals through NOTCH receptors on stalk cells to maintain the stalk cell phenotype [53,54]. Our results are also different than those recently observed suggesting exosomes from mesenchymal stem cells (MSC) with miR-30b overexpression or inhibition positively or negatively affected endothelial cell tube formation respectively [55]. However in this study, the authors do not indicate that altered miR-30b levels specifically, as transferred to HUVECs from MSC exosomes, is responsible for the increased tube formation noted. No data was provided to show that modulating miR-30b in MSC does not lead to additional changes in the miRNA or protein content of the exosomes that could account for or contribute to the changes in tube formation seen in their system. Despite this, our data, along with these additional findings, indicate the importance of controlled miR-30b expression to angiogenesis, with our data specifically highlighting miR-30b as a VEGF-regulated miRNA with anti-angiogenic potential.

Seeking to identify potential targets that could mediate the observed inhibition of miR-30b overexpression on endothelial capillary morphogenesis, we identified up-regulation of TGFβ2 in response to overexpressing miR-30b. Knockout mouse models have shown the importance of TGFβ signaling during embryonic vascular development with deletion of ligands TGFβ1 and TGFβ2 as well as receptors TGFβR1 and TGFβR2 all exhibiting developmental defects often leading to embryonic lethality [56–59]. In certain contexts, TGFβs have been shown to negatively regulate processes important for angiogenesis [39,40,60]. Specifically in regards to capillary morphogenesis, TGFβ1 has been shown to impede capillary-like cord formation in bovine microvascular endothelial cells [41]. Interestingly, our results indicate that TGFβ2, but not TGFβ1, is suppressed by VEGF in HUVECs and that capillary morphogenesis is inhibited by TGFβ2 in these cells, specifically highlighting TGFβ2 as anti-angiogenic in our system. Interestingly, it has been recently shown that KSHV infection of endothelial cells also downregulates TGFβ2 [61], and given that KSHV upregulates VEGF, our proposed pathway of VEGF-regulation of miR-30b and its downstream effects on TGFβ2 is also supported by this finding. Importantly, our data indicates that cells with higher levels of miR-30b secrete more TGFβ2 and that these cells have higher levels of Smad2 phosphorylation indicating an active autocrine TGFβ signaling pathway. As opposed to signaling through ALK1 and Smad1/5 which results in promotion of endothelial proliferation and migration, TGFβ signaling through TGFβR1 and Smad2/3 mediates inhibitory signals in endothelium [39], thus our result indicating increased Smad2 activation fits with established literature regarding the inhibitory effects of signaling pathways stimulated by TGFβs in endothelial cells. Confirming the importance of TGFβ2 in inhibiting capillary morphogenesis regulated by miR-30b expression, use of TGFβ2 neutralizing antibodies restored capillary-like cord formation in miR-30b overexpressing cells, suggesting an autocrine signaling function for TGFβ2 in regulation of capillary morphogenesis. Autocrine TGFβ signaling through Smad2 in cultured HUVECs has been previously observed [62]. Thus, our results fit a model whereby miR-30b increases levels of secreted TGFβ2 which functions in an autocrine signaling pathway to mediate the inhibitory effects of increased miR-30b expression on endothelial cell capillary morphogenesis.

As TGFβ2 is increased with miR-30b overexpression it cannot be a direct target of this miRNA. We thus speculated that miR-30b targets a repressor of TGFβ2 expression. It has been previously suggested that miR-21 directly targets the 3’ UTR of TGFβ2 leading to its degradation and hence suppresses its expression [63], and in our array, we did observe upregulation of miR-21 following VEGF stimulation of HUVEC (Fig 1A). While it is true that this upregulation of miR-21 following VEGF stimulation of endothelial cells may also contribute to promoting angiogenesis by downregulation of TGFβ2 which inhibits this process, the increased expression of TGFβ2 observed following miR-30b overexpression observed in our experiments is independent of miR-21, as we did not see any significant differences in miR-21 levels in HUVEC transfected with miR-30b mimics as compared to controls (S1 Fig). As such, we speculated that the target of miR-30b could be a protein involved in TGFβ2 transcription. ATF2 has been shown to be a direct transcriptional activator of the TGFβ2 gene [42], and we showed that ATF2 negatively impacted endothelial cell capillary morphogenesis as its depletion enhanced capillary-like cord formation in HUVECs. Phosphorylation of ATF2 on residues Thr69 and Thr71 is required for its dimerization and activation [64], and we have shown that increased activation of ATF2 occurs in response to high levels of miR-30b and that ATF2 depletion inhibits the induction of TGFβ2 expression by miR-30b, confirming a requirement for ATF2 in mediating the effects of miR-30b on TGFβ2 expression. Our data suggests that enhanced ATF2 activation results from miR-30b targeting of a known ATF2 repressor, namely JDP2 [36] in cells overexpressing miR-30b. We demonstrated a direct effect of miR-30b on JDP2 levels in HUVEC and JDP2 is a predicted target of miR-30b containing a miR-30b binding site in its 3’-UTR [assessed by TargetScan (http://www.targetscan.org)]. JDP2 has been shown to directly bind to ATF2 in a stable heterodimer on CRE-element containing promoter sequences [36], and this interaction prevents ATF2 mediated gene transcription. We speculate that reduced JDP2 allows ATF2 to form other hetero- or homodimers that are subsequently activated via JNK/p38 kinases as has been previously shown [65–67], resulting in the increased phosphorylation of ATF2 as we observe. As such, our data suggest a possible mechanism whereby miR-30b targets JDP2 thus freeing ATF2 from repression and promoting its activation and binding to promoter sequences such as in the TGFβ2 gene. In turn, the anti-angiogenic effects of miR-30b are manifested by the subsequent autocrine inhibitory effects of TGFβ2 in endothelial cells. These autocrine inhibitory effects are overcome by VEGF stimulation resulting in decreased TGFβ2 levels in part via downregulation of miR-30b.

## Conclusions

Understanding mechanistically how individual miRNAs function to regulate complex processes such as capillary morphogenesis is important; however, context-specific functions of miRNAs need to be considered. Furthering our understanding of VEGF-mediated miRNA expression in angiogenic processes, we have identified miR-30b as a VEGF regulated miRNA with a negative regulatory role in capillary morphogenesis of HUVEC. Importantly, we identified the mechanism of miR-30b inhibition of capillary morphogenesis via regulation of an autocrine inhibition manifested by increased TGFβ2 production. We have also shown that miR-30b influences TGFβ2 production through regulation of the activity of the TGFβ2 transcription factor ATF2, via targeting one of its known repressors. Thus, our results make an important link and further elucidate a mechanism of cross-regulation between the pro-angiogenic growth factor VEGF and TGFβ2 expression in endothelial cells and highlight the importance of autocrine growth factor signaling regulated by miRNAs, to angiogenic processes.

## Supporting information

S1 Fig MiR-21 expression levels do not change following modulation of miR-30b levels with specific mimic and inhibitor. HUVEC were transfected with control or miR-30b mimic (20 nM) and control or miR-30b inhibitor (50 nM) and expression of miR-21 was assessed at 48 hours post transfection. Endogenous control used for normalization was miR-103. Data represents the mean ± SEM (n = 2).
(TIF)

S1 Table Affymetrix miRNA 1.0 chip expression results. (XLS)

## Abbreviations

ATF2: Activating transcription factor 2
BME: Basement membrane extract
ELISA: Enzyme-linked immunosorbent assay
FBS: Fetal bovine serum
HUVEC: Human umbilical vein endothelial cell
JDP2: Jun dimerization protein 2
miRNA: MicroRNA
mRNA: Messenger RNA
MSC: Mesenchymal Stem Cell
qRT-PCR: Quantitative reverse transcription polymerase chain reaction
siRNA: Small interfering RNA
TGFβ: Transforming growth factor beta
TGFβR: TGFβ receptor
VEGF: Vascular endothelial growth factor
VEGFR: VEGF receptor
