# Supplementary figures and images for: MicroRNA-30b controls endothelial cell capillary morphogenesis through regulation of transforming growth factor beta 2
Source: PLoS One. 2017 Oct 4;12(10):e0185619. doi: 10.1371/journal.pone.0185619 (PMC5627931; doi:10.1371/journal.pone.0185619)

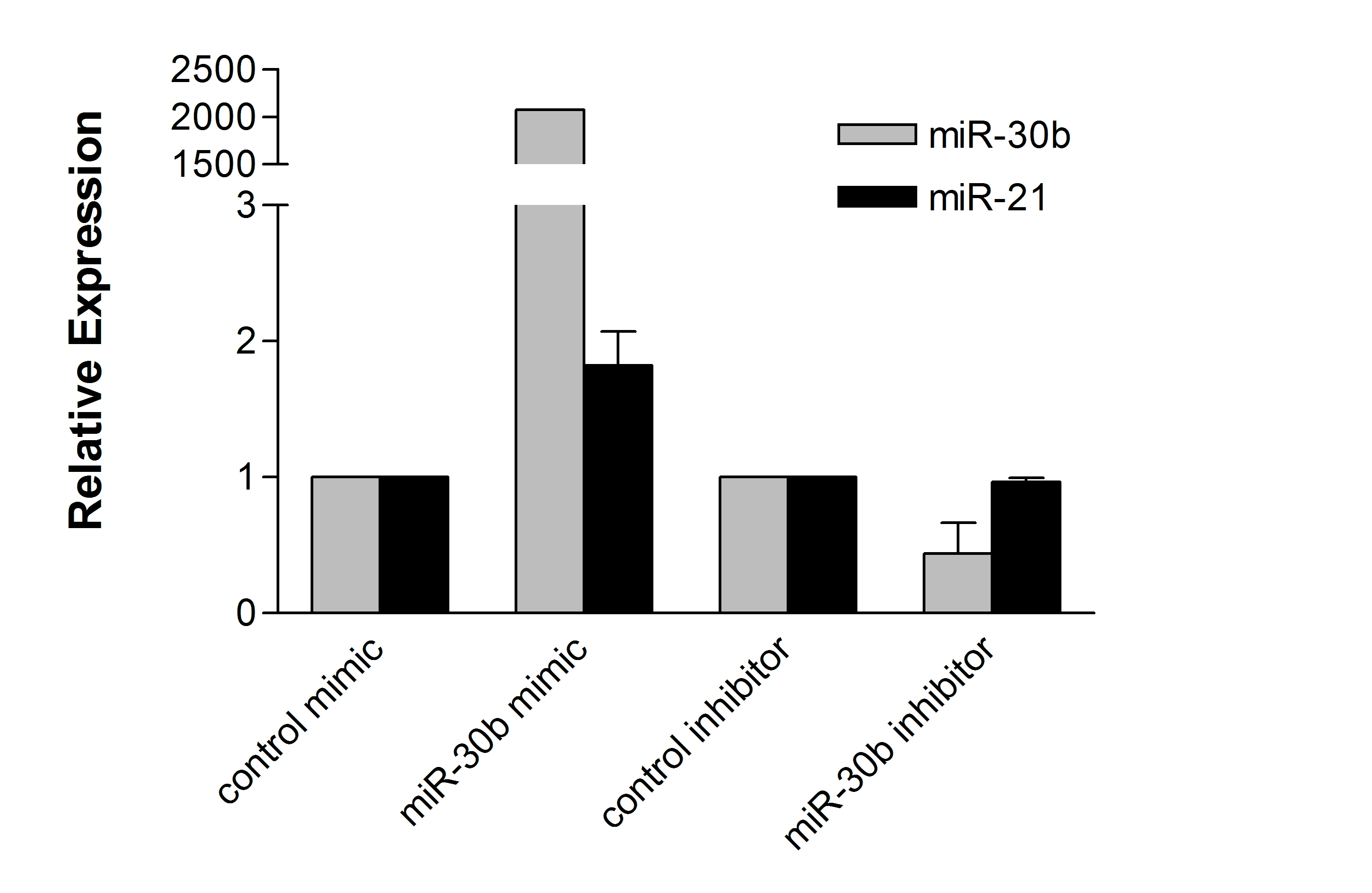

Supplement: S1 Fig — HUVEC were transfected with control or miR-30b mimic (20 nM) and control or miR-30b inhibitor (50 nM) and expression of miR-21 was assessed at 48 hours post transfection. Endogenous control used for normalization was miR-103. Data represents the mean ± SEM (n = 2). (TIF) [file pone.0185619.s001.tif]
